# Antimicrobial activity of a new class of synthetic retinoid antibiotics and comparator agents against ocular staphylococci

**DOI:** 10.3389/frabi.2023.1101450

**Published:** 2023-03-22

**Authors:** Camille André, Cassandra L. Schrank, Ana Victoria Cheng Jaramillo, Eleftherios Mylonakis, William M. Wuest, Michael S. Gilmore, Wooseong Kim, Paulo J. M. Bispo

**Affiliations:** ^1^ Department of Ophthalmology, Massachusetts Eye and Ear, Harvard Medical School, Boston, MA, United States; ^2^ Infectious Disease Institute, Massachusetts Eye and Ear, Harvard Medical School, Boston, MA, United States; ^3^ CIRI - Centre International de Recherche en Infectiologie, Inserm, U1111, Université Claude Bernard Lyon 1, CNRS, UMR5308, Ecole Normale Supérieure de Lyon, Lyon, France; ^4^ Department of Chemistry, Emory University, Atlanta, GA, United States; ^5^ Infectious Diseases Division, Warren Alpert Medical School of Brown University, Providence, RI, United States; ^6^ Department of Microbiology and Immunobiology, Harvard Medical School, Boston, MA, United States; ^7^ College of Pharmacy, Graduate School of Pharmaceutical Sciences, Ewha Womans University, Seoul, Republic of Korea

**Keywords:** retinoids, Staphylococcus aureus, Coagulase-negative staphylococci, multidrug-resistance, antimicrobial resistance (AMR), ocular infections

## Abstract

**Objectives:**

Antimicrobial resistance is global pandemic that poses a major threat to vision health as ocular pathogens, especially staphylococcal species, are becoming increasingly resistant to first-line therapies. Here we evaluated the antimicrobial activity of a new class of synthetic retinoids in comparison to currently used antibiotics against clinically relevant ocular staphylococcal isolates.

**Methods:**

Antimicrobial susceptibility testing was performed by broth microdilution for 3 novel synthetic retinoids (CD1530, CD437, and a CD437 analogue) and 7 comparator antibiotics, against a collection of 216 clinical isolates.

**Results:**

CD437 MIC_50_ and MIC_90_ were 2 µg/mL for *Staphylococcus aureus*, and 1 µg/mL and 2 µg/mL respectively, for coagulase-negative staphylococci (CoNS). CD1530 (MIC_50_ = 2 µg/mL for all species) also displayed good activity with an *in vitro* potency slightly lower (2-fold) for *S. aureus* (MIC_90_ = 4 µg/mL) when compared to CD437. A CD437 analogue also demonstrated good *in vitro* activity (MIC_50_ = 2 µg/mL for all species) and potency (MIC_90_ = 2 µg/mL for MRSA and 4 µg/mL for MSSA and CoNS). *In vitro* potencies were similar or higher than that of comparator agents, and were not impacted by multidrug resistance phenotypes.

**Conclusion:**

Our results demonstrate that synthetic retinoids display potent *in vitro* activity against ocular staphylococcal species, including multidrug-resistant isolates.

## Introduction

Bacteria are the most common etiologies of eye infections, with Gram positive organisms such as *Staphylococcus aureus* and Coagulase-Negative Staphylococci (CoNS) being among the leading causes of serious and sight-threatening diseases (O’Callaghan, 2018; Tanaka et al., 2019; Kowalski et al., 2020). Empirical treatment with a broad-spectrum antimicrobial agent is the mainstream approach to control these infections. However, because of the emergence of antimicrobial resistance (AMR) in ophthalmology, these approaches are becoming increasingly compromised (Bispo et al., 2022). Staphylococcal isolates display the highest resistance levels among ocular bacteria and are becoming highly resistant to first-line topical therapies (Thomas et al., 2019; Asbell et al., 2020). In the United States, about 40% of ocular *S. aureus* isolates and 49% of CoNS collected through a nationwide surveillance program are resistant to methicillin (Asbell et al., 2020). Methicillin resistance is a key phenotype in *S. aureus* and CoNS that is associated with increased co-resistance rates to first-line antimicrobials used in ophthalmology (Asbell et al., 2020).

Increasing multidrug resistance (MDR) in methicillin-resistant isolates is further compounded by their propensity to form non-growing subpopulations of persister cells that exhibit high levels of intrinsic tolerance to antibiotics, and have a role in chronic and recurrent infections (Lew and Waldvogel, 2004). This phenomenon happens in all *S. aureus* cultures growing at high densities and is common in staphylococcal biofilms (Dombach et al., 2021), a mode of growth that is associated with a variety of ocular infections (Bispo et al., 2015). As current antibiotic therapies are not effective against persister cells and biofilms, and are increasingly compromised by the global emergence of AMR, the development of novel therapeutics that circumvent these limitations are urgently needed.

We recently found that the membrane-targeting synthetic retinoid molecules CD437, CD1540 and a second generation CD437 analogue (named Analog 2), have potent *in vitro* activity against methicillin-resistant *S. aureus* (MRSA) and their persister cells and biofilms, and show a low probability of resistance development (Kim et al., 2018). These advantages motivated us to explore synthetic retinoids as potential novel therapies to treat recalcitrant sight-threatening infections. Here, we report the pre-clinical results on the *in vitro* activity of CD437, CD1530, Analog 2 and comparator antimicrobials against a large collection of ocular staphylococci isolates.

## Materials and methods

### Bacterial isolates

Protocols for obtaining bacterial isolates collected for infection diagnosis were approved by the MEE Institutional Review Board (IRB). Since this study only included discarded bacterial isolates de-identified that were frozen in our pathogen repository, written informed consent was waived by the MEE IRB. Primary clinical specimens collected from multiple ocular infection sites were obtained by the attending ophthalmologist or resident following institutional guidelines and submitted to the clinical microbiology laboratory for processing. In total, 216 non-duplicate and consecutive staphylococcal isolates collected from patients presenting from 2014 to 2017 at MEE with keratitis (n=111) endophthalmitis (n=30), conjunctivitis (n=19), orbital abscess (n=17) and cellulitis (n=15) and dacryocystitis (n=14) were included. Bacterial identification was performed using the MicroScan WalkAway system (Beckman Coulter). Frozen isolates stored at -80°C were recovered on 5% sheep blood agar plates (BD Biosciences) and incubated overnight at 37°C.

### Antimicrobial susceptibility testing

Antimicrobial susceptibility testing was performed by broth microdilution following the CLSI recommendations, using cation-supplemented Mueller-Hinton broth (CA-MHB) and 96-well plates (Greiner Bio-One, Inc., Longwood, FL) (Weinstein et al., 2015). The minimum inhibitory concentration (MIC) value for each antibiotic was determined as the lowest concentration of antibiotic that inhibited bacterial growth as visualized by the naked eye. Each clinical isolate was tested *in vitro* against three novel synthetic retinoid antibiotics including CD437, CD1530, and CD437 analogue (Analog 2). Comparator antibiotics that are commonly used for treatment of ocular bacterial infections were also tested including besifloxacin, moxifloxacin, levofloxacin, ofloxacin, tobramycin, azithromycin and vancomycin. Quality assurance was performed by concurrently testing control strains: MRSA MW2 (Baba et al., 2002) and *S. aureus* ATCC 29213. Interpretive criteria published by CLSI were applied for the comparator antibiotics (Clinical and Laboratory Standards Institute, 2018). CD437 and CD1530 were acquired from R&D Systems (Minneapolis, MN, USA). Analog 2 was synthesized and tested as previously described (Kim et al., 2018). MIC_50_ and MIC_90_ were calculated using SPSS software (version 24, IBM, Armonk, New York).

## Results

In this study, we evaluated the *in vitro* activity of 3 novel synthetic retinoid antibiotics (CD437, CD1530, Analog 2) and comparator antimicrobial agents against 216 ocular staphylococci isolates, including 174 *S. aureus* (of which 56 were MRSA), and 42 CoNS species.

All retinoid molecules were active *in vitro* against this population of ocular staphylococci isolates (**Table 1**). The MIC_50_ and MIC_90_ for CD437 was 2 µg/mL for methicillin-susceptible *S. aureus* (MSSA) and MRSA, and 1 µg/mL and 2 µg/mL, respectively, for CoNS. CD1530 (MIC_50_ 2 µg/mL for all species) also displayed potent activity against ocular staphylococci with an *in vitro* potency slightly lower (2-fold) for *S. aureus* (MIC_90_ 4 µg/mL) when compared to CD437 (MIC_90_ 2 µg/mL). Analog 2 also demonstrated good *in vitro* activity (MIC_50_ 2 µg/mL for all species) and potency (MIC_90_ 2 µg/mL for MRSA and 4 µg/mL for MSSA and CoNS).

**Table 1:** Antimicrobial activity of retinoids tested against staphylococci isolates causing ocular infections at Massachusetts Eye and Ear Infirmary.

Organism/Retinoid compound	No. of isolates and cumulative % inhibited at MIC (µg/mL) of								MIC (µg/mL)	
	≤ 0.12	0.25	0.5	1	2	4	8	16	MIC_50_	MIC_90_
MSSA (n=118)										
CD437				22 (18.6)	87 (92.4)	9 (100)			2	2
CD1530					65 (55.1)	53 (100)			2	4
Analogue2	1 (0.8)			2 (2.5)	97 (84.7)	16 (98.3)	1 (99.2)	1 (100)	2	4
MRSA (n=56)										
CD437				6 (10.7)	48 (96.4)	2 (100)			2	2
CD1530				2 (3.6)	34 (64.3)	20 (100)			2	4
Analog 2		1 (1.8)		1 (3.6)	50 (92.9)	4 (100)			2	2
Coagulase negative staphylococci (n=42)										
CD437		2 (4.8)	5 (16.7)	23 (71.4)	10 (95.2)	2 (100)			1	2
CD1530		2 (4.8)	5 (16.7)	5 (28.6)	29 (97.6)	1 (100)			2	2
Analogue2		2 (4.8)	1 (7.1)	8 (26.2)	22 (78.6)	8 (97.6)	1 (100)		2	4

Almost half of the MSSA isolates (47.5%) were resistant to azithromycin and approximately 16% of them were resistant to fluoroquinolones (using ofloxacin as the class substrate - **Table 2**). The *in vitro* potency of the retinoids was higher (2 to 4-fold) for MSSA (MIC_90_ 2 or 4 µg/mL) compared to that of ofloxacin (MIC_90_ 8 µg/mL), and was similar to levofloxacin and moxifloxacin (MIC_90_ 2 µg/mL). Resistance to non-β-lactam antibiotics was high among MRSA isolates, with 32.1% of the isolates being resistant to tobramycin (MIC_90_ >16 µg/mL), 60.7% to the fluoroquinolones (MIC_90_ >32 µg/mL for ofloxacin and levofloxacin, 8 µg/mL for moxifloxacin and 2 µg/mL for besifloxacin), and 92.9% to azithromycin (MIC_90_ >16 µg/mL). Around 29% of ocular CoNS isolates were resistant to fluoroquinolones and almost half of them were resistant to azithromycin (42.9%). The *in vitro* potency of the retinoids was higher against MRSA and CoNS when compared to ofloxacin and levofloxacin (at least 8 to 16-fold), moxifloxacin (2 to 4-fold) and to azithromycin (at least 4 to 8-fold). Retinoids were also more potent than tobramycin against MRSA isolates (at least 4 to 8-fold). *In vitro* potency of all retinoids against MRSA (MIC_90s_ 2 or 4 µg/mL) was similar to that of the newest ophthalmic fluoroquinolone agent, besifloxacin (MIC_90_ 2 µg/mL).

**Table 2:** Comparative antimicrobial activity of retinoids and comparator agents tested against staphylococci isolates causing ocular infections at Massachusetts Eye and Ear Infirmary.

Organism/resistance group (no. tested/antimicrobial agent)	MIC (µg/mL)			CLSI, %S/%NS
	MIC_50_	MIC_90_	Range	
MSSA (n=118)				
CD437	2	2	1-4	
CD1530	2	4	2-4	
Analog 2	2	4	≤0.12-16	
Besifloxacin	≤0.25	≤0.5	≤0.25-8	
Moxifloxacin	≤0.25	2	≤0.25->32	87.3/12.7
Levofloxacin	≤0.25	2	≤0.25->32	85.6/14.4
Ofloxacin	0.5	8	≤0.25->32	83.9/16.1
Tobramycin	0.25	0.5	≤0.12->16	95.8/4.2
Azithromycin	2	>16	≤.012->16	52.5/47.5
Vancomycin	1	1	0.25-2	100.0/0.0
MRSA (n=56)				
CD437	2	2	1-4	
CD1530	2	4	1-4	
Analog 2	2	2	0.25-4	
Besifloxacin	≤0.25	2	≤0.25-8	
Moxifloxacin	1	8	≤0.25->32	44.6/55.4
Levofloxacin	4	>32	≤0.25->32	39.3/60.7
Ofloxacin	4	>32	≤0.25->32	39.3/60.7
Tobramycin	0.5	>16	≤.012->16	67.9/32.1
Azithromycin	>16	>16	≤.012->16	7.1/92.9
Vancomycin	1	2	0.25-2	100.0/0.0
CoNS (n=42)				
CD437	1	2	0.25-4	
CD1530	2	2	0.25-4	
Analog 2	2	4	0.25-8	
Besifloxacin	≤0.25	0.5	≤0.25-8	
Moxifloxacin	≤0.25	8	≤0.25-32	78.6/21.4
Levofloxacin	≤0.25	>32	≤0.25->32	71.4/28.6
Ofloxacin	0.5	>32	≤0.25->32	71.4/28.6
Tobramycin	≤0.12	1	≤0.12->16	92.9/7.1
Azithromycin	1	>16	≤0.12->16	57.1/42.9
Vancomycin	2	2	0.25-4	100.0/0.0

S, susceptible; NS, non susceptible.

All isolates were susceptible to vancomycin. Retinoids *in vitro* potency was slightly lower for MSSA (MIC_90_ 2 and 4 µg/mL) when compared to vancomycin (MIC_90_ 1 µg/mL). For MRSA, the *in vitro* potency was the same as that of vancomycin (MIC_90_ 2 µg/mL), except for CD1530 (MIC_90_ 4 µg/mL). For CoNS isolates, CD437 and CD1530 *in vitro* potency was the same as vancomycin (MIC_90_ 2 µg/mL), whereas Analogue2 *in vitro* potency was slightly lower (MIC_90_ 4 µg/mL).

## Discussion

Staphylococcal species are the leading causes of ocular infections, including serious and sight-threatening diseases such as infectious keratitis and endophthalmitis (O’Callaghan, 2018; Tanaka et al., 2019; Kowalski et al., 2020; Bispo et al., 2022). Nationwide multicenter surveillance studies show increasing and remarkably high antibiotic resistance levels among ocular pathogens, especially for staphylococcal species (Thomas et al., 2019; Asbell et al., 2020; Bispo et al., 2022). Fluoroquinolones are the most commonly used antibiotics to prevent and treat these infections, and its overuse has resulted in the emergence of high levels of resistance to many molecules in the class, especially among methicillin-resistant ocular staphylococci species (Haas et al., 2011; Asbell et al., 2020). In our collection of isolates, approximately 60% of all methicillin-resistant staphylococci were resistant to the fluoroquinolones, a rate of resistance that is similar to what has been found in large-scale surveillance studies. For example, data generated by the Antibiotic Resistance Monitoring in Ocular Microorganisms (ARMOR) study have shown that approximately 70% of the ocular MRSA isolates (n=765) collected from 2009 to 2018 in the United States were resistant to at least one fluoroquinolone antibiotic (Asbell et al., 2020). Vancomycin has also been used in ophthalmology for decades and remains the primary option when ocular infections are known or suspected to be caused by MDR strains of *S. aureus* and CoNS (Lin et al., 2019). Although staphylococci remain largely sensitive to vancomycin, after decades of heavy use in general medicine, intermediate vancomycin resistance has been increasingly encountered (Dao et al., 2020). In addition, vancomycin has a fairly weak bactericidal activity for staphylococci (Sakoulas et al., 2004). The new synthetic retinoids tested in our study have exhibited potent *in vitro* activity against MDR MRSA strains and are known to also be active against their subpopulations of persiter cells and biofilms (Kim et al., 2018). With a view towards developing the next-generation of improved therapies to treat recalcitrant ocular staphylococcal infections, we began to perform pre-clinical studies on the *in vitro* activity of these novel synthetic retinoids against a large collection of ocular staphylococcal isolates.

Our results indicate that retinoids represent a potential option for the treatment of ocular infections caused by staphylococci species, including isolates that are now highly resistant to antimicrobials commonly used clinically. The emergence of AMR in ophthalmology and particularly among ocular staphylococci species highlights the need for the development of novel therapies that are efficient against resistant organisms and that can prevent selection of resistant subpopulations in the ocular environment. Retinoids are narrow-spectrum drugs active against Gram-positive bacteria only and with low probability of resistance selection (Kim et al., 2018). Serial passages of MRSA for 100 days in sub-MIC levels of CD437 yielded only putative mutants with 2-fold increase in the MIC, while parallel passage in ciprofloxacin resulted in the selection of subpopulations that were 256-fold more resistant (Kim et al., 2018). While membrane-acting antimicrobials are often cytotoxic to mammalian cells, CD437 and CD1530 are relatively non-toxic (Kim et al., 2018). These molecules are amenable to further structural modifications in their backbone and branch groups that can improve their cytotoxic profiles (and water solubility), while maintaining good antimicrobial activity (Kim et al., 2018; Cheng et al., 2020; Haney et al., 2021). Analog 2 for example, is a modified version of CD437 that is significantly less toxic to human cells *in vitro* (Kim et al., 2018). This analogue synthetic retinoid retained the same bactericidal profile of CD437 against MRSA, their persisters and biofilms (Kim et al., 2018), and has shown to be highly active against our population of staphylococci from ocular infections.

In summary, this preclinical study demonstrated that synthetic retinoids display potent *in vitro* activity against ocular staphylococci species including MDR isolates, with potencies that are similar to vancomycin, and higher than other comparator agents currently used as standard of care for treatment of ocular infections, especially when compared to fluoroquinolones versus MRSA and CoNS, and to azithromycin. Antimicrobial resistance is a global pandemic that poses a major threat to human health and is especially important in ophthalmology, as delays in effective treatment of eye infections and failures due to AMR can result in irreversible damage of key sensory functions. Further experiments, including *ex vivo* and *in vivo* experiments are needed to understand the drug penetration in the eye. Synthetic retinoids are potential options that can be developed into improved local therapies for hard-to-treat and recalcitrant ocular infections caused by increasingly resistant staphylococci species.

## Data availability statement

The raw data supporting the conclusions of this article will be made available by the authors, without undue reservation.

## Ethics statement

Protocols for obtaining bacterial isolates collected for infection diagnosis were approved by the MEE Institutional Review Board (IRB). Since this study only included discarded bacterial isolates de-identified that were frozen in our pathogen repository, written informed consent was waived by the MEE IRB.

## Author contributions

CA, PB, CS, AJ, EM, WW, MG, and WK contributed to conception and design of the study. CA and PB organized the database. CS, AJ, EM, WW, MG, WK provided materials. CA and PB wrote the first draft of the manuscript. All authors contributed to the article and approved the submitted version.
